# Effects of Teleintervention Maintenance Programs for Children (Age 6–12) and Adolescents (Age 13–18) With Overweight or Obesity—Promoting a Healthy Lifestyle: A Systematic Review

**DOI:** 10.1111/ijpo.70158

**Published:** 2026-09-27

**Authors:** Anna Wittmann, Pia Späth, Feline Zocher, Eckard Nagel, Michael Lauerer, Magdalena Schellenberg

**Affiliations:** ^1^ Institute for Medical Management and Health Sciences University of Bayreuth Bayreuth Germany; ^2^ Deutsche Rentenversicherung Nordbayern Bayreuth Germany

**Keywords:** digital aftercare, maintenance phase, paediatric obesity, post‐rehabilitation intervention, teleintervention programs, weight management

## Abstract

**Background:**

Weight management and maintenance of healthy habits are crucial in combating paediatric obesity. This systematic review examines outcomes beyond BMI in teleintervention maintenance programmes, with a focus on sustaining a healthy lifestyle.

**Methods:**

A PubMed, LIVIVO and Web of Science search was conducted for publications between 2012 and 4 November 2025 to identify primary studies evaluating teleintervention maintenance programmes of at least 12 weeks' duration, delivered to children and adolescents with overweight or obesity following structured obesity treatment. We extracted healthy lifestyle parameters (e.g., quality of life), behaviour change (e.g., diet) and clinician‐reported outcomes (e.g., BMI). Quality assessment was tailored to study type. As data did not permit meta‐analysis, we performed a narrative synthesis.

**Results:**

Screening 2543 articles yielded 6 RCTs and 2 pre‐post studies. Findings highlight improvements in psychosocial outcomes (e.g., social well‐being), lifestyle modifications in daily choices (e.g., dietary patterns) and behaviours (e.g., screen time), and statistically significant outcomes including reductions in body fat percentage, metabolic profile improvements, cardiovascular enhancements and BMI stabilisation. Feasibility insights were also gained.

**Conclusion:**

Teleintervention maintenance programmes following structured paediatric obesity treatment were associated with stabilisation of previously achieved weight‐related and behavioural gains, rather than greater weight loss than comparator care, alongside consistently high feasibility and substantial reductions in healthcare visits and cost. Given the heterogeneity of designs and outcomes, findings should be interpreted as directional rather than pooled effect estimates, and a human component may be necessary to sustain them.

## 1 Introduction

Paediatric obesity is one of the most serious public health challenges of the 21st century and is now recognised as a global pandemic [1]. Children and adolescents with obesity encounter several physical problems such as sleep apnoea, asthma, orthopaedic problems [2], linked with an increased risk for many health complications (e.g., type 2 diabetes, hypertension and heart disease) [3]. Additionally, in the short term, paediatric overweight is intricately associated with stigmatisation and psychosocial distress, manifesting in various ways such as diminished self‐esteem and heightened symptoms of anxiety and depressive disorders [4, 5]. Children with overweight or obesity often encounter societal biases and negative perceptions, which are associated with feelings of isolation and inadequacy [4]. These health implications are also associated with lower school participation, attainment and educational performance, as well as with an increased risk of unemployment in adulthood and reduced life expectancy [6]. Considered in its complexity as a chronic disease, obesity presents a multifaceted challenge and requires effective treatment strategies which should start as early as possible [1].

While structured lifestyle interventions for children and adolescents with obesity have demonstrated initial success in reducing body weight and improving comorbidities, the intended long‐term effects on body mass index (BMI) remain negligible [7]. Achieving a lasting impact therefore requires ongoing and consistent efforts beyond the initial treatment phase [8], ideally ensuring continuity of care from childhood through the transition to adult services [9]. Post‐rehabilitation interventions, hereafter referred to as aftercare or the maintenance phase, consequently represent a critical yet comparatively under‐examined component of paediatric obesity treatment.

At the same time, there is growing public health consensus that treatment success should not be evaluated by weight‐related metrics alone. Research on weight stigmatisation indicates that an exclusive focus on weight loss, particularly when internalised by the individual, may harm physical and psychosocial health and reduce quality of life [10]. Social factors further shape eating habits and physical activity levels and are therefore central to long‐term treatment success. While anthropometric measures such as BMI remain necessary clinical indicators, psychosocial and behavioural outcomes, including quality of life and health behaviours, constitute complementary criteria of sustained treatment success.

Recently, telehealth weight management interventions for treating paediatric obesity have been developed, demonstrating good feasibility and acceptability [11] and can enhance the well‐being of children [12]. Clinical practice and research in teleinterventions for paediatric weight management are dynamic and evolving [13], particularly given their potential to reduce barriers to care, improve access, and support long‐term weight management. Most of all, the growing emphasis on digital health solutions also creates a strong foundation for more patient‐centred obesity treatment approaches [14].

Prior evidence syntheses only partially address this field. A meta‐analysis by van der Heijden et al. [8] demonstrated small positive effects of maintenance interventions on anthropometric outcomes in children with overweight or obesity. However, that review (a) was not specific to tele‐delivered aftercare, (b) predates the rapid expansion of digital health following 2018 and, in particular, the COVID‐19 pandemic and (c) focused primarily on weight‐related endpoints. Furthermore, a recent review examined digital strategies in paediatric weight management [15] but did not systematically address the post‐treatment maintenance phase. To our knowledge, no systematic review has yet synthesised the evidence on teleintervention aftercare programmes following structured paediatric obesity treatment with a systematic focus on outcomes beyond weight‐related metrics, such as psychosocial well‐being and health behaviours. The present review addresses this gap.

To ensure conceptual clarity, in this review, *aftercare*, the maintenance phase (in the German rehabilitation context referred to as aftercare/Nachsorge), is defined as follow‐up support delivered after completion of an initial structured obesity treatment, with the goal of sustaining behavioural and health gains over time. In line with our eligibility criteria, aftercare interventions were required to last at least 12 weeks and to be delivered beyond the original intensive treatment period. We considered *teleintervention* to describe aftercare delivered primarily through digital or remote modalities (e.g., SMS, telephone calls, mobile applications, web‐based programmes, or wearable devices), either as fully automated support or as blended models that include a human component (e.g., clinicians and case managers) and may involve parents/caregivers.

Using these definitions, we examined outcomes in three domains: the primary outcomes of (1) healthy‐lifestyle parameters (e.g., quality of life and psychosocial well‐being) and (2) behaviour change (e.g., diet, physical activity and screen time) and the secondary outcome of (3) clinician‐reported outcomes (e.g., BMI‐SDS and metabolic parameters) as they remain relevant indicators for obesity.

## 2 Materials and Methods

The review question was structured according to the PICO framework: children and adolescents aged 6–18 years with overweight or obesity who had completed a structured obesity treatment programme (P); telehealth maintenance (aftercare) programmes of at least 12 weeks' duration (I); usual care with or without structured follow‐up, no aftercare, or pre‐intervention values in uncontrolled studies (C); healthy lifestyle parameters and behaviour change as primary outcomes and clinician‐reported outcomes as secondary outcomes (O).

### 2.1 Search Strategy

To answer the research question, the PubMed, LIVIVO and Web of Science databases were systematically searched for corresponding studies. We followed the Preferred Reporting Items for Systematic Reviews and Meta‐analyses (PRISMA) [16] guidelines (see Table S1) and meeting inclusion from 2012 through November 4, 2025, as prespecified and documented in the research protocol (PROSPERO registration number CRD42023487963 [17]). When the review was initiated in 2022, aftercare had become a prominent topic within the German healthcare system; therefore, a 10‐year retrospective period was selected to capture the relevant developments in this field. Briefly, included search terms related to ‘youth’ or ‘adolescents’ with ‘overweight’ or ‘obesity’, as well as ‘telemedicine’ and ‘aftercare’ and related terms. The search algorithm is in Table S2. To identify further potential studies, we also conducted a targeted search on www.connectedpapers.com.

### 2.2 Eligibility Criteria

Only primary studies were eligible. To capture the range of available evidence on maintenance interventions delivered as teleintervention programmes following a structured obesity treatment, both randomised controlled trials and uncontrolled before–after designs were included. Eligible studies had to report at least one outcome beyond weight‐related metrics, reflecting the review's focus on the wider health and well‐being of children and adolescents with overweight or obesity.

### 2.3 Inclusion and Exclusion Criteria

To be included, articles had to report primary studies with a primary or secondary outcome of healthy lifestyle parameters and behaviour change (Table S3).

Inclusion criteria consisted of (1) examining children and adolescents aged 6–18 years, based on the PubMed MeSH definition of children (6–12 years) and adolescents (13–18 years); (2) diagnosed with overweight or obesity defined with BMI ≥ 25, or BMI ≥ p85 for age, or BMI z‐score > + 1SD; (3) following an initial structured treatment programme for overweight or obesity; (4) aftercare defined as ‘maintenance phase’ and lasting for at least 12 weeks delivered as teleintervention programme; (5) the intervention must involve technology (e.g., phone calls, application, online programme), rather than as a temporary adaptation to pandemic‐related restrictions; (6) the studies reported at least one primary outcome (healthy‐lifestyle parameter or behaviour change) in addition to a weight‐related metric; (7) were written in the English or German language and published in a peer‐reviewed journal; (8) when children and adolescents were addressed as a family in a community‐based setting receiving the aftercare intervention, data could be presented separately for children and adolescents to examine the effect on children; and (9) primary studies using a randomised controlled or uncontrolled before–after (pre–post) design.

Papers were excluded when (1) focusing on the primary prevention of overweight with normal‐weight children or adolescents, (2) examining children and adolescents with overweight or obesity due to a secondary or syndromic cause, (3) the intervention was used as an adjunct tool to support in‐person sessions or clinical visits for regular treatment, (4) parents were the target group of the aftercare intervention without addressing children and adolescents separately, or when (5) the target population was exclusively infants or adults (i.e., < 6 or > 18 years old). Further, studies were excluded in case (6) the study was not retrievable even after contacting the authors. Finally, (7) protocol papers, qualitative studies, case reports, conference abstracts and systematic reviews were also excluded.

### 2.4 Outcomes

In line with the aim of examining outcomes beyond weight‐related metrics, the primary outcomes were healthy‐lifestyle parameters (quality of life and psychosocial well‐being) and behaviour change (dietary behaviour, physical activity and screen time). Secondary outcomes were clinician‐reported outcomes, comprising weight‐related metrics (BMI z‐score/BMI‐SDS, or BMI where no adjusted metric was reported), metabolic and cardiovascular parameters and body composition. Studies were additionally required to report at least one primary outcome alongside a weight‐related metric (eligibility criterion 6). Process outcomes relating to implementation, from self‐monitoring and engagement with the intervention tools, feasibility, adherence and acceptability, were extracted where reported, as these inform the interpretation of effectiveness findings but were not treated as outcomes of effectiveness.

### 2.5 Study Selection and Data Extraction

**FIGURE 1:**
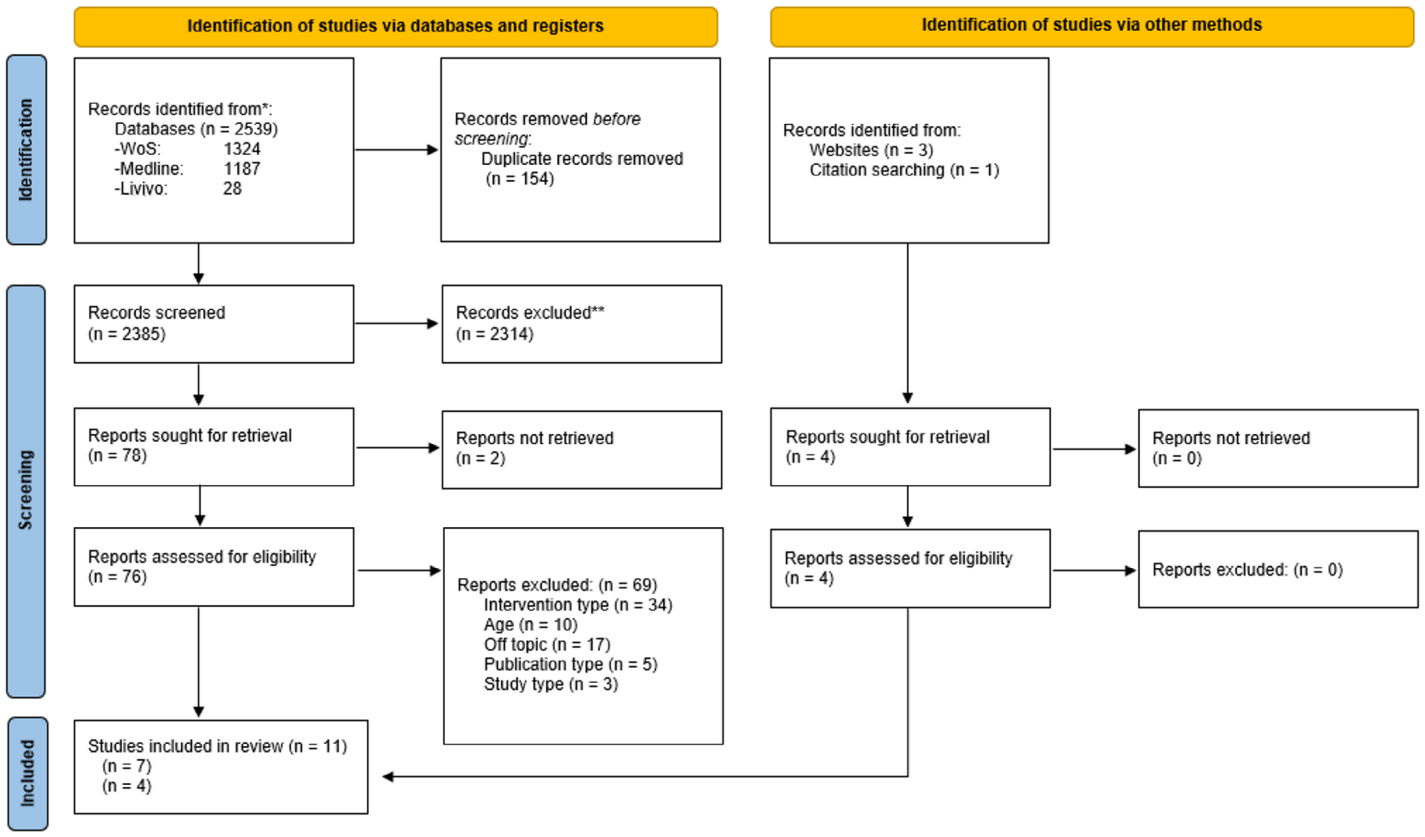
PRISMA flow chart summarising the identification, screening, and inclusion of studies.

Search results were exported to Citavi and screened automatically for duplicates. Titles and abstracts were then screened independently against the eligibility criteria by three authors (AW, FZ and PS). Records considered potentially eligible were retrieved in full and assessed independently by four reviewers (AW, FZ, MS and PS). Disagreements at both stages were resolved through consensus discussion among the reviewers, and reasons for excluding full‐text records were documented (Table S4). Where articles reported identical participants or cohorts, the most recent article was presented in the Results. The flow of study selection is presented in the PRISMA diagram (Figure 1).

Data were extracted independently by four researchers (AW, FZ, MS and PS), strengthening the reliability of the extracted study data, including author, year of publication, study design, type of technology used, length of intervention, participant characteristics, outcomes and results, supported by Sabrina Seidenfad as acknowledged. Four reviewers (AW, FZ, MS and PS) independently assessed the risk of bias of the included studies, with the tool selected according to study design. For randomised controlled trials, the ROB2 tool [18] was used, with overall bias scored as ‘high risk’, ‘some concerns’ or ‘low risk’. For uncontrolled before–after (pre–post) studies, the NIH quality‐assessment tool for before–after studies with no control group [19] was used, with overall quality rated ‘good’, ‘fair’ or ‘poor’. Disagreements about grading were resolved through consensus discussion. Disagreements about study inclusion or grading among the four independent raters were resolved through consensus discussion.

### 2.6 Data Synthesis

As pre‐specified in the protocol (PROSPERO CRD42023487963), meta‐analysis was planned where at least two studies reported the same outcome using comparable designs, comparators and instruments. These conditions were not met for any outcome: the included studies showed substantial heterogeneity in delivery mode, comparators, outcome instruments and follow‐up timing. Eating behaviour, for instance, was assessed with four different instruments across four studies and physical activity ranged from self‐report to accelerometry. We therefore conducted a structured narrative synthesis in line with the synthesis without meta‐analysis [20].

For each outcome, we summarised the number of studies reporting a statistically significant effect in the hypothesised direction relative to the total number of studies assessing that outcome, without inferring pooled effect magnitudes.

The outcomes reported in this systematic review were examined for similarities and clustered around five topics, accordingly:

- Behaviour change (dietary patterns, physical activity and sedentary time).
- Psychosocial outcomes (quality of life and emotional well‐being).
- Self‐Management behaviours (adherence to self‐monitoring).
- Technology use (feasibility, adherence and satisfaction of intervention).
- Health outcomes (blood pressure, metabolic profile, physical fitness, body fat percentage and waist circumference/WtHR (waist to height ratio), BMI).

These five clusters operationalise the outcome domains specified above: clusters (a) and (c) correspond to behaviour change, clusters (b) and (d) to healthy‐lifestyle parameters and cluster (e) to clinician‐reported outcomes.

## 3 Results

### 3.1 Intervention Characteristics

Database searching resulted in a total of 2539 records, with four additional records from other sources. After removing 154 duplicates, 2385 records were screened, of which 2314 were excluded. Of 76 full‐text articles assessed, 11 publications met the inclusion criteria. Six publications reported three studies: Garza et al. [21] and Rhyu and Park [22]; Chen et al. [23, 24]; and Nguyen et al. [25, 26]. Therefore, these 11 publications correspond to 8 unique studies: 6 randomised controlled trials and 2 uncontrolled before–after studies without a control group. The selection process is shown in the PRISMA flow diagram (Figure 1).

The characteristics of the eight included studies, together with a description of the maintenance (aftercare) phase delivered after the initial intensive treatment, are presented in Table 1. The outcome data are reported by domain in Tables 2, 3, 4, 5, 6, 7, 8.

**TABLE 1:** Characteristics of the included studies and description of the maintenance (aftercare) phase.

Study (ref)	Country/setting	*N* (analysed)	Design	Population (age; inclusion)	Initial (intensive) treatment	Maintenance phase (delivery, dose, duration)	Comparator
Garza et al. [21]./Rhyu and Park [22]	USA; community (Hispanic families)	71/56	RCT (3 arms)	Overweight/obese children; family‐based 10–14 years	8‐week residential summer camp (diet, exercise, education)	10‐month telephone aftercare: weekly 10–20 min calls by a single trained investigator; pedometer self‐monitoring (goal 10 000 steps/day), parent‐reported weekly	Camp only (no aftercare); and no‐treatment control
Chen et al. [23, 24]	USA; outpatient	40	RCT	Overweight/obese adolescents 13–18 years	3‐month self‐monitoring with Fitbit flex + iStart Smart online modules	Biweekly text messages reinforcing healthy‐lifestyle maintenance during the 3‐month maintenance phase; continued Fitbit use	Usual care (control group)
Nguyen et al. [25, 26] (Loozit)	Australia; hospital + community	151	RCT	Overweight/obese adolescents 13–16 years	2‐month intensive group lifestyle programme	Additional therapeutic contact (telephone coaching + email/SMS) over ~24 months versus group programme alone	Group programme alone (no additional contact)
Abraham et al. [27]	Hong Kong; tertiary clinic	48	RCT (3 arms)	Obese adolescents 12–18 years	Usual care visits with a physician (baseline, midpoint, end)	12‐week internet‐based curriculum + weekly cell‐phone/SMS reminders over 6 months	Usual care; and simplified lifestyle‐modification (nutrition‐counselling) arm
Schiel et al. [28]	Germany; inpatient rehabilitation	61	RCT	Overweight/obese children and adolescents (mean 14.4 years)	6‐week inpatient rehabilitation	12‐month telemedical aftercare: case‐manager contact via SMS/email at defined intervals; sensor‐based accelerometry to set individual activity goals	Standard care (no telemedical support)
Foissac et al. [29]	France; tertiary hospital	78	RCT	Adolescents 11–17 years with severe obesity (BMI‐z 3.95)	3‐month intensive family‐based multidisciplinary intervention	15‐month app‐only remote monitoring (self‐monitoring, goal‐setting, automated feedback); caregivers had no app access, no scheduled visits	Traditional monitoring (quarterly face‐to‐face multidisciplinary visits)
Straker et al. [30]. (CAFAP)	Australia; community	69	Uncontrolled pre‐post (waitlist)	Overweight/obese adolescents (mean 14.1 years)	8‐week intensive family‐centered programme (exercise, diet, behaviour)	12‐month telephone + text‐message maintenance support at decreasing frequency	Waitlist (within‐subject) and no concurrent control group
Jensen et al. [31]	USA; outpatient	16	Uncontrolled pre‐post (single arm)	Overweight/obese adolescents, 13–17 years	12‐week in‐person behavioural treatment + smartphone self‐monitoring	12‐week electronic‐only maintenance phase: daily text messages + smartphone self‐monitoring app, no in‐person contact	None (single‐arm pilot)

*Note:* Studies are presented in the order used throughout the Results. Garza et al. and Rhyu and Park and the two Nguyen et al. reports and the two Chen et al. reports, each report a single. Unique study; the 11 included publications therefore correspond to 8 unique studies (6 randomised controlled trials and 2 uncontrolled before–after studies). The ‘maintenance phase’ column describes the aftercare intervention delivered after completion of the initial intensive treatment, which was the focus of this review. Note on group labelling: comparator groups are described by their actual content (usual care, group programme alone, traditional face‐to‐face monitoring, waitlist, or none) rather than by a generic ‘after‐intervention’ label, to make the nature of each comparison explicit.

Abbreviations: CT, randomised controlled trial; *N*, number of participants analysed.

**TABLE 2:** Main findings of diet‐related outcomes.

Study	*N*	Follow‐up	Instrument	Direction	Finding (exact values)
Chen et al.	40	6 months	Self‐reported dietary intake (California Health Interview Survey items)	↑/↓	Intervention group showed a significant increase in fruit and vegetable servings/day (z = 2.74, *p* = 0.006) and a significant decrease in soda/sweetened‐drink servings/day (z = −3.19, *p* = 0.001) vs. control at 6 months.
Nguyen et al.	151	24 months	15‐item food‐frequency questionnaire + eating‐behaviour questions (dichotomised, GEE)	↓/↑	Pre–post (both arms combined): less frequent high‐fat meat products (OR 0.22, 95% CI 0.14–0.36) and daily lunch (OR 0.49, 0.30–0.82); more likely to never/rarely consume fruit juice (OR 2.47, 1.59–3.82). Additional therapeutic contact conferred no further benefit.
Straker et al.	69	12 months	3‐day food records (servings of fruit, vegetables, junk food; negative binomial regression)	↑/↓/↔	During intervention vs. waitlist: fruit servings increased (monthly IRR 1.33, 95% CI 1.11–1.60, *p* = 0.004) and junk‐food servings decreased (IRR 0.83, 0.74–0.94, *p* = 0.020); vegetable servings did not change significantly (IRR 1.00). Fruit and junk‐food changes were largely maintained at 12 months.
Foissac et al.	78	15 months	Study‐specific 16‐item eating‐behaviour score (lower = better)	↔ (IG)	Eating behaviour improved significantly in the control group (8.6 → 7.6, *p* = 0.02) but not in the intervention group (8.7 → 8.1, *p* = 0.18); no between‐group difference. Lower scores indicate better eating behaviour.

*Note:* Abraham et al. is not included here because it assessed a dietary‐knowledge score (max 75), not dietary intake and reported no significant between‐ or within‐group change; it belongs in the knowledge/other‐outcomes table rather than the diet‐intake table. Direction: ↑ increase, ↓ decrease, ↔ no significant change. Directional statements refer to the intervention group unless otherwise noted; for Foissac et al., lower eating‐behaviour scores indicate better eating behaviour Chen et al. and Nguyen et al. each report one unique study.

Abbreviations: CG, control group; CI, confidence interval; IG, intervention group; IRR, incidence rate ratio; OR, odds ratio.

**TABLE 3:** Main findings of physical activity outcomes.

Study	*N*	Follow‐up	Instrument	Direction	Finding (exact values)
Chen et al.	40	6 months	Self‐reported physical activity and screen time (California Health Interview Survey items)	↑/↓	Significant increase in physical‐activity days/week (z = 2.58, *p* = 0.01) and significant decrease in TV/computer time (z = −3.34, *p* = 0.001) in the intervention group vs. control at 6 months.
Nguyen et al.	151	24 months	Children's Leisure Activities Study Survey (CLASS), self‐report	↓/↑	Pre–post (both arms combined): light‐intensity physical activity decreased (−0.80 h/week, 95% CI −0.96 to −0.64); total leisure activities increased (+ 1.2 h, 1.0–1.4), including non‐screen‐based (+ 1.4 h). At 12 months, screen time decreased (−0.8 h, *p* = 0.045). The additional therapeutic contact conferred no benefit and was associated with more computer time at 24 months.
Straker et al.	69	12 months	Actical accelerometers (sedentary, light, moderate, vigorous activity)	↑/↓/↔	During intervention vs. waitlist: moderate physical activity increased (+ 1.8 min/day/month, *p* = 0.041) and sedentary time decreased (−5.1 min/day/month, *p* = 0.014). Light and vigorous activity unchanged. Changes largely maintained across the 12‐month maintenance period.
Foissac et al.	78	15 months	Self‐reported physical and sedentary activity (h/week)	↔	No significant change in physical activity (control 13.9 → 16, *p* = 0.42; intervention 13.8 → 15.8, *p* = 0.13) or sedentary activity (control 22.4 → 18.7, *p* = 0.24; intervention 17.1 → 16.4, *p* = 0.72); no between‐group difference.
Schiel et al.	61	12 months	Sensor‐based mobile‐phone accelerometry (DiaTrace, Fraunhofer IGD); movement patterns validated (CV 2.67% walking, 2.84% running)	n.r.	Physical activity was measured objectively and used to derive individual activity goals during telemedical aftercare, but changes in physical activity over the 12‐month aftercare period are not reported; outcomes are limited to BMI and BMI‐SDS.

*Note:* Garza et al./Rhyu and Park used pedometers and Jensen et al. a smartphone app to track activity, but in both cases these served as self‐monitoring tools rather than reported outcomes; the corresponding adherence data are presented in Table 4. Abraham et al. measured a self‐reported physical‐activity score (max 10) with no significant change. Direction: ↑ increase, ↓ decrease, ↔ no significant change, Directional statements refer to the intervention group unless otherwise noted. Chen et al. and Nguyen et al. each report one unique study.

Abbreviations: CV, coefficient of variation; n.r., measured but not reported.

**TABLE 4:** Main findings of psychosocial indicators.

Study	*N*	Follow‐up	Instrument	Direction	Finding (exact values)
Chen et al.	40	6 months	Health Behaviour Questionnaire self‐efficacy subscales (physical‐activity and dietary self‐efficacy)	↑	Intervention group showed a significant increase in dietary self‐efficacy (z = 5.05, *p* = 0.001; large effect) and physical‐activity self‐efficacy (*z* = 2.75, *p* = 0.006) vs. control at 6 months.
Nguyen et al.	151	24 months	Mental Health Inventory‐5, body‐dissatisfaction scale, MacArthur subjective social status, Harter Self‐Perception Profile	↑	Pre–post (both arms combined): improvements in body‐shape satisfaction (+0.43, 95% CI 0.20–0.65), subjective social status (+1.26, 0.86–1.66), and global self‐worth (+0.20, 0.09–0.32), with most Harter self‐perception domains improving (except close friendship). Additional therapeutic contact conferred no benefit (and was associated with lower perceived athletic competence).
Foissac et al.	78	15 months	Paediatric Quality of Life Inventory (PedsQL 4.0), self‐ and parent‐report	↑/↔	Self‐reported total PedsQL improved significantly in both arms (traditional 74 → 80.3, *p* < 0.001; remote 71.7 → 77.5, *p* < 0.001), with emotional and social subscales improving in the remote arm; however, there was no significant between‐group difference. Parent‐reported total PedsQL did not change significantly. Parent–child concordance was fair at baseline (ICC 0.48) and poor at end (ICC 0.24).

*Note:* Straker et al. collected psychosocial and quality‐of‐life data but reported them in a separate publication, so no psychosocial outcomes are extractable from the included report. Abraham et al. measured depression, anxiety and stress (DASS‐21) and reported no significant between‐ or within‐group change; these are summarised in the other‐outcomes table. Direction: ↑ increase (improvement), ↓ decrease, ↔ no significant change. Directional statements refer to the intervention group unless otherwise noted. Chen et al. and Nguyen et al. each report one unique study.

Abbreviation: ICC, intraclass correlation coefficient.

**TABLE 5:** Main findings for a healthy lifestyle.

Study	*N*	Follow‐up	Measure	Finding (exact values)
Chen et al.	40	6 months	Fitbit Flex app use; provider data‐sharing (self‐reported)	75% accessed the tracking app several times/week and 20% weekly; 100% found the device helpful for tracking physical activity, 88% for food intake; 91% shared Fitbit data with their primary‐care provider.
Nguyen et al.	151	24 months	Booster‐session attendance; telephone‐coaching and SMS/email uptake	Booster‐session attendance declined from 69% to 31% between first and final session; adolescents received a median of 12 telephone‐coaching sessions and 31 SMS/email messages, with a median reply rate of only 12%.
Abraham et al.	48	6 months	Internet‐curriculum completion; SMS response rate	87.5% of intervention adolescents read the curriculum and 71% completed all lessons, but only 4 parents read it; 400 SMS were sent, with response rates of 78.3% (dietary goals) and 77.5% (exercise goals).
Jensen et al.	16	3 months (electronic‐only)	Smartphone self‐monitoring of diet and physical activity (DailyBurn Tracker)	Diet self‐monitoring fell from 48.3% of in‐person‐phase days to 16.6% of electronic‐only‐phase days, and physical‐activity self‐monitoring from 14.6% to 4.6%; both declines were statistically significant (*t* (15)=5.68, *p* < 0.001, *d* = 0.46; *t* (15)=3.67, *p* = 0.002, *d* = 0.38). Self‐monitoring was not significantly correlated with change in zBMI. This is the only included study to test engagement decline statistically.
Schiel et al.	61	12 months	Case‐manager‐supported telemedical monitoring (SMS/email contact at defined intervals)	A case‐manager (psychologist) maintained contact via SMS/email and reviewed monitoring data, initiating direct contact if targets (activity, calorie intake, psychological concerns) were not met. High treatment adherence is reported as a strength of the intervention, but adherence was not quantified.
Straker et al.	69	12 months	Telephone/text maintenance support; in‐session self‐monitoring	Adolescents were taught to self‐monitor heart rate and perceived exertion. Maintenance support was delivered by telephone and text at decreasing frequency. Quantified adherence to the text/phone component was not reported; attrition was high (69 entered, 34 completed).
Garza et al./Rhyu and Park	71/56	10 months	Pedometer‐based self‐monitoring; weekly parent‐reported step counts	Each aftercare participant received a pedometer with a goal of 10 000 steps/day (encouraged, not required); parents reported weekly step readings during telephone calls. Adherence was not quantified, and the authors note that self‐reported diet and step data may not have been accurate.
Foissac et al.	78	15 months	Mobile‐application usage	Adherence to regular application use was not recorded; only self‐reported usage frequency was noted and these data were not analysed, so users and non‐users could not be compared.

*Note:* This cluster captures self‐management behaviours (self‐monitoring, tool use and engagement) rather than significance‐tested health outcomes; values are descriptive adherence metrics except where a statistical test is reported (Jensen et al.). Chen et al., Nguyen et al. and Garza/Rhyu each report one unique study. Cross‐study observation: initial uptake of self‐monitoring was high (75%–87.5%), but sustained engagement was consistently lower. Only one study tested this decline statistically, showing that self‐monitoring fell significantly once human contact was withdrawn (Jensen et al.: diet 48.3% → 16.6%, physical activity 14.6% → 4.6%). Three studies did not quantify adherence to the self‐monitoring component at all (Straker; Garza/Rhyu; Foissac), and one reported it descriptively without analysis (Schiel), limiting conclusions about the relationship between engagement dose and outcomes.

**TABLE 6:** Main outcomes for the practicality of teleintervention aftercare programs.

Study	*N*	Follow‐up	Measure	Finding (exact values)
Chen et al.	40	6 months	Feasibility, acceptability, retention	Adolescents rated the mobile intervention positively and 100% would recommend it to others. Retention was high (90% intervention, 87% control). Delivery required ~2 h/week of research‐assistant time for personalised reminders.
Nguyen et al.	151	24 months	Program satisfaction, deliverability, safety	87% of adolescents and 97% of parents would recommend the program. The additional therapeutic contact was feasible to deliver but provided no added benefit. Recorded adverse events included parent‐reported disordered eating (*n* = 3) and poor body image (*n* = 2).
Abraham et al.	48	6 months	Feasibility (primary outcome), acceptability	Feasibility was the primary outcome: high recruitment, retention and log‐in rates. 100% of intervention students rated the weekly SMS useful or very useful, 81.3% the curriculum, and 100% goal‐setting. ~2 h/week of research‐assistant time was needed for reminders. Authors concluded the approach is feasible but effectiveness on weight remains unproven.
Jensen et al.	16	3 months (electronic‐only)	Satisfaction (Client Satisfaction Questionnaire); exit interview	Overall satisfaction was high (CSQ 20.33 of a maximum 22). However, 73.3% of participants stated they preferred meeting in a group over the electronic‐only intervention; 53.3% found the self‐monitoring app ‘tedious’ and ‘difficult to use’; 73.3% viewed the text messages positively (described as ‘enthusiastic’ and ‘uplifting’), while 13.3% said they ‘didn't get as much help as [they] would have liked’.
Schiel et al.	61	12 months	Adherence, subjective acceptance	High treatment adherence and a positive subjective experience of the telemedical intervention are reported as key strengths. The authors identify group inhomogeneity arising from group randomisation as a principal weakness, and note markedly unequal re‐examination rates (79% intervention vs. 41% control).
Garza et al./Rhyu and Park	71/56	10 months	Deliverability; measurement validity	Telephone aftercare was delivered as weekly calls of 10–20 min by a single trained investigator over 10 months and maintained camp‐derived improvements. The authors note that adherence to diet and step goals relied on unverified parent/child self‐report, precluding strict control of intervention dose.
Straker et al.	69	12 months	Not formally assessed	No formal feasibility or acceptability measure was reported; a process evaluation exploring barriers to completion was underway at the time of publication. Community delivery by health professionals was described as challenging but achievable, and program fidelity was assessed by independent observation. No adverse events were reported.
Foissac et al.	78	15 months	Compliance, healthcare‐resource use, cost	Compliance was high (92.3%). Remote monitoring significantly reduced follow‐up visits during the maintenance phase (median 5 → 2; *p* < 0.0001) and was approximately 2.5× less expensive per patient per year than traditional monitoring, with equivalent weight control.

*Note:* Values are descriptive feasibility/acceptability metrics rather than significance‐tested effect estimates, except where a statistical comparison is reported (Foissac et al. visit reduction, *p* < 0.0001). Cross‐study observation: acceptability was consistently high where formally assessed (87%–100% would recommend; CSQ 20.33/22), but three findings temper this. First, feasibility was resource‐dependent: three studies reported substantial staff input (~2 h/week of research‐assistant time in Chen and Abraham; weekly 10–20‐min calls by a single investigator in Garza/Rhyu). Second, high global satisfaction coexisted with a clear preference for human contact—73.3% of participants preferred group meetings over the electronic‐only phase and 53.3% found the self‐monitoring app burdensome (Jensen et al.). Third, the most clearly quantified benefit was reduced healthcare burden and cost rather than improved clinical outcomes (Foissac et al.).

**TABLE 7:** Main outcomes for health outcomes.

Study	*N*	Follow‐up	Instrument	Direction	Finding (exact values)
Garza^a^ et al./Rhyu^a^ and Park	71/56	10 months	Lipid profile (TC, TG, LDL‐C, HDL‐C); VO_2_max; body fat % (BIA); inflammatory and oxidative‐stress markers	↓/↑ maintained	The 8‐week camp improved lipids, fitness and body fat in both camp arms. The aftercare effect was maintenance at 10 months: the camp+telephone arm retained improvements vs. baseline (total cholesterol *p* = 0.036; triglycerides *p* = 0.024; LDL‐C *p* = 0.029; HDL‐C *p* = 0.036; VO_2_max *p* < 0.01; body fat *p* = 0.021/0.016), whereas the camp‐only arm reverted (body fat POST→1YEAR *p* = 0.017; VO_2_max *p* = 0.011). Between‐arm difference at 1YEAR in body fat (*p* = 0.037) and flexibility (*p* = 0.037). Rhyu reports maintained improvements in inflammatory and oxidative‐stress markers in the telephone arm.
Chen et al.	40	6 months	Blood pressure; waist‐to‐hip ratio	↓/↔	Significant decrease in diastolic blood pressure in the intervention group vs. control (*z* = −3.23, *p* = 0.001). Systolic blood pressure (*p* = 0.97) and waist‐to‐hip ratio (*p* = 0.47) unchanged.
Nguyen et al.	151	24 months	Fasting lipids, glucose, insulin, ALT; blood pressure; waist circumference, waist: height ratio	↓/↑/↔	Pre–post (both arms combined): total cholesterol ↓ (−0.2 mmol/L, 95% CI −0.3 to −0.1) and triglycerides ↓ (ratio 0.9); HDL cholesterol also ↓ (−0.1; unfavourable). Systolic (+4 mmHg) and diastolic (+2 mmHg) blood pressure increased, within the normal range. LDL, glucose, insulin, ALT unchanged. Waist: height ratio ↓ (−0.02, −0.03 to −0.01); waist circumference unchanged. The telephone/electronic adjunct conferred no benefit.
Abraham et al.	48	6 months	Blood pressure; body fat (%); waist and hip circumference	↔	No consistent significant differences between or within groups. Borderline decreases in systolic (*p* = 0.046) and diastolic (*p* = 0.047) blood pressure occurred in the nutritional‐counselling arm only, not the internet/text arm. Body fat, waist and hip circumference unchanged (all *p* > 0.05).
Schiel et al.	61	12 months	Carotid intima‐media thickness; 24‐h ambulatory blood pressure; body composition (BIA); fasting glucose, oGTT, lipids, TSH, uric acid, CRP	↔ (baseline/discharge only)	Between‐group comparisons showed no significant differences in carotid intima‐media thickness (0.56 ± 0.09 vs. 0.56 ± 0.08 mm, *p* = 0.93) or 24‐h blood pressure (systolic 136.9 ± 12.7 vs. 133.2 ± 10.7 mmHg, *p* = 0.28; diastolic 76.5 ± 6.9 vs. 79.1 ± 9.8 mmHg, *p* = 0.26). Body fat at discharge did not differ (33.9% ± 5.3% vs. 36.4% ± 5.8%, *p* = 0.11), though fat‐free mass did (59.4 ± 10.5 vs. 46.9 ± 12.0 kg, *p* = 0.001), reflecting baseline group differences. Laboratory parameters were comparable at baseline except LDL cholesterol (2.89 ± 0.83 vs. 2.49 ± 0.57 mmol/L, *p* = 0.04). Changes in these parameters over the 12‐month aftercare period are not reported.
Foissac et al.	78	15 months	Metabolic syndrome panel (dyslipidaemia, insulin resistance, steatosis)	↔	No significant change for dyslipidaemia (*p* = 0.99), severe insulin resistance (*p* = 0.57), biological steatosis (*p* = 0.08), radiological steatosis (*p* = 0.99) or hepatic steatosis (*p* = 0.99); no between‐group differences. The authors state that metabolic improvements could not be demonstrated.

*Note:* Direction: ↑ increase · ↓ decrease · ↔ no significant change. Directional statements refer to the aftercare/intervention arm unless otherwise noted; for Nguyen et al., values are pre–post changes for both arms combined. Straker et al. and Jensen et al. reported no metabolic, cardiovascular, or body‐composition outcomes other than BMI z‐score, which is presented with the weight‐related outcomes (Table 7). Cross‐study observation: the clearest aftercare effect was maintenance rather than further improvement—in the only study with a no‐aftercare comparator, telephone aftercare preserved lipid, fitness and body‐fat gains that reverted without it (Garza/Rhyu). Elsewhere, metabolic and cardiovascular outcomes were largely unchanged: one study showed a diastolic blood‐pressure reduction (Chen), one showed mixed changes including an unfavourable HDL decrease (Nguyen), and two showed predominantly null findings (Abraham, Foissac). One study assessed a comprehensive metabolic and cardiovascular panel but reported only baseline and discharge comparisons, not change over the aftercare period (Schiel).

Abbreviations: ALT, alanine aminotransferase; BIA, bioelectrical impedance analysis; oGTT, oral glucose tolerance test; TC, total cholesterol; TG, triglycerides.

^a^ Studies use identical participants.

**TABLE 8:** Main outcomes for weight‐related outcomes.

Study	*N*	Aftercare duration	Weight metric	Direction	Finding (exact values)
Garza et al./Rhyu and Park (one study, two reports)	71/56	10 months (telephone)	BMI (kg/m^2^)^a^	↓ maintained	BMI fell during the 8‐week camp in both camp arms (PITI 27.6 → 26.5, *p* < 0.01; PI 27.5 → 26.7, *p* < 0.01). At 10 months the camp + telephone arm retained the reduction (26.8; below baseline *p* < 0.05, and significantly different from the camp‐only arm), whereas the camp‐only arm returned to baseline (27.7). Control BMI increased (27.8 → 28.7, *p* < 0.05). Rhyu reports the same pattern (SUTI 27.2 → 26.1 → 26.4 vs. SU 27.0 → 26.2 → 27.2).
Chen et al.	40	6 months	BMI z‐score	↓	Significant time × group effect favouring the intervention (coefficient−0.12, SE 0.03, *z* = −4.36, *p* = 0.001; effect size *d* = 0.34): BMI z‐score decreased in the intervention group relative to control over 6 months.
Nguyen et al.	151	24 months	BMI z‐score	↓ (no adjunct effect)	Pre–post (both arms combined): BMI z‐score fell from 2.02/2.03 at baseline to 1.93/1.83 at 24 months (mean change −0.13, 95% CI −0.20 to −0.06). Relative to the group program alone, the additional therapeutic contact (telephone/SMS/email) had no impact on BMI z‐score.
Abraham et al.	48	6 months	BMI (kg/m^2^)^a^	↔	No significant differences in BMI between or within the internet/text, nutritional‐counselling and control groups over 24 weeks (1st visit *p* = 0.142; 2nd visit *p* = 0.065; baseline *p* = 0.032 reflects a group imbalance at entry). Post hoc observed power for BMI was 0.122.
Schiel et al.	61	12 months	BMI (kg/m^2^) and BMI‐SDS^b^	↑ (partially maintained)	Both groups lost weight during the 6‐week in‐house rehabilitation (IG BMI 33.8 → 31.4, *p* < 0.001; BMI‐SDS 2.70 → 2.43, *p* < 0.001; CG BMI 30.8 → 29.2, *p* = 0.43; BMI‐SDS 2.53 → 2.31, *p* = 0.001). Over the 12‐month telemedical follow‐up both groups regained weight, but only the intervention group remained significantly below its own baseline (BMI 32.7, *p* = 0.001; BMI‐SDS 2.59, *p* = 0.002 vs. trial onset), whereas the control group did not (BMI 30.7, *p* = 0.69; BMI‐SDS 2.51, *p* = 0.29).^b^
Foissac et al.	78	15 months	BMI z‐score (primary: reduction > 0.5 SD)	↔	No significant difference in success rate between remote (31.6%) and traditional (25.0%) monitoring (*p* = 0.69); OR 1.49 (95% CI 0.53–4.20). Mean BMI z‐score change −0.21 (SD 0.44) remote vs. −0.19 (0.47) traditional (*p* = 0.87). Severe obesity (BMI z ≥ 4 SD) fell from 51.4% to 40% (remote) and 48.6% to 37.8% (traditional), with no between‐group difference.
Straker et al.	69	12 months	BMI z‐score	↓ then ↔	BMI z‐score did not change significantly during the waitlist or intervention periods. During maintenance, point estimates were significantly below pre‐intervention (2.11, SE 0.02) at 3 months (2.05, *p* = 0.035) and 6 months (2.03, *p* = 0.042), but no longer at 12 months (2.03, *p* = 0.060). Rates of change did not differ between waitlist, intervention and maintenance periods.
Jensen et al.	16	3 months (electronic‐only)	BMI z‐score (zBMI)	↓ then reverted	zBMI decreased significantly over the 12‐week in‐person + smartphone phase (−0.08 SD units; *t* (13) = 2.22, *p* = 0.04, *d* = 0.63; 1.85 → 1.74). During the subsequent 12‐week electronic‐only maintenance phase, gains were NOT maintained: neither time 3 nor time 4 zBMI differed significantly from baseline (1.78; 1.78). Self‐monitoring fell from ~50% of combined‐intervention days to < 20% of electronic‐only days. The authors conclude smartphones may be less effective for maintaining gains after intensive treatment.

*Note:* Direction: ↓ decrease, ↑ increase, ↔ no significant change. Directional statements refer to the aftercare/intervention arm unless otherwise noted; for Nguyen et al., values are pre–post changes for both arms combined. BMI z‐score/BMI‐SDS is reported as the primary weight metric because raw BMI is confounded by age and sex in growing children. Cross‐study observation: on the age‐ and sex‐adjusted metric, only one study demonstrated a weight benefit attributable to the teleintervention relative to a comparator (Chen et al.: BMI z‐score time × group *p* = 0.001); Nguyen et al. found a pre–post reduction across both arms with no added benefit from the telephone/electronic adjunct, Straker et al. found improvements that were no longer significant at 12 months, and Foissac et al. found no difference between remote and traditional monitoring. The most informative contrast concerns the delivery mode of maintenance: aftercare involving continued human contact preserved gains achieved during intensive treatment (Garza/Rhyu, telephone; Schiel, telemedical support with clinician contact), whereas an electronic‐only maintenance phase did not, with treatment gains reverting to baseline as self‐monitoring adherence fell below 20% (Jensen et al.). Taken together, the evidence supports teleintervention aftercare as a means of stabilising weight‐related gains rather than producing greater weight loss than comparator care, and suggests that a human component may be necessary for that stabilisation.

Abbreviations: CG, control group; IG, intervention group.

^a^ Raw BMI is reported for Garza et al./Rhyu and Park and Abraham et al. because these studies did not report BMI z‐scores or BMI‐SDS. As raw BMI is not adjusted for age and sex, and participants grew in height over follow‐up, these values should be interpreted with caution and are not directly comparable with the BMI z‐score data from other studies.

^b^ Schiel et al.: comparisons are within‐group versus trial onset, not between groups. The groups were not comparable at baseline (IG BMI 33.8 vs. CG 30.8) and re‐examination rates differed markedly (79% IG vs. 41% CG), which should be considered when interpreting these findings.

The studies were conducted across five countries: the United States, Australia, Germany, France and Hong Kong, with in outpatient, community, tertiary‐care and inpatient‐rehabilitation settings and comprised six randomised controlled trials and two uncontrolled before–after studies. Sample sizes ranged from 16 to 151 participants aged 11–18 years, and the maintenance phase lasted between 3 and 24 months. Aftercare was delivered through a range of modalities: from telephone calls, text messages, internet curricula, mobile applications and wearable or pedometer‐based self‐monitoring and in most studies followed a structured intensive intervention such as an inpatient rehabilitation stay, a residential camp, or an intensive family‐based programme.

### 3.2 Setting, Follow‐Up and Population

All studies shared similar settings in tertiary care or community‐based environments. Participants had overweight or obesity and initially underwent a structured treatment designed for paediatric obesity (minimum 6 weeks), followed by a weight maintenance treatment to support individuals for a minimum of 12 weeks to sustain a healthy lifestyle and ensure continued success. Follow‐up of the aftercare intervention was 6 months [23, 24, 27, 31], 12 months [21, 22, 25, 28, 29, 30] and 24 months [26]. The average age of subjects ranged from 10 to 18 years old (mean: 13.85), with a sample size ranging from 40 to 151 in the RCTs and 16 to 68 in the Pre‐post studies. One study [25, 26] had a sample size greater than 100 participants. Three studies were conducted in the USA (Garza et al. [21]/Rhyu et al. [22]; Chen et al. [23, 24]; Jensen et al. [31]), two studies in Australia (Nguyen et al. [25, 26]; Straker et al. [30]), one in Germany (Schiel et al. [28].), France (Foissac et al. [29]) and in Hong Kong (Abraham et al. [27]). All studies were published between 2012 and 2025. Ten articles were written in English, and one paper in German.

### 3.3 Type of Maintenance‐Intervention

#### 3.3.1 Intervention Content

Most maintenance interventions primarily focused on transferring knowledge related to health topics, with a particular emphasis on nutrition and physical and leisure activity. Additionally, behavioural change strategies and motivational elements were important aspects of the intervention content to address various aspects of participants' lifestyle choices and well‐being by teaching emotional regulation or learning relaxation techniques. The strategies in promoting awareness of the participants' lifestyle in the studies ranged from traditional methods like telephone calls offering guidance on adhering to diet and physical activity to more contemporary strategies and actively engaging with the lifestyle by involving technology for tracking and monitoring by mobile devices or commercially available smartphone apps. Building on this, in the majority of studies, a human component was integrated to communicate directly with the participants with the exception of three studies (Chen et al. [23, 24], Foissac et al. [29] and Jensen et al. [31]). In these three studies, participants received text messages from study staff but could not respond to them.

Regarding the field of weight management in paediatric obesity, the studies extended beyond the individual efforts of the children and included the broader family dynamic.

Most studies involved family members, typically a parent or caregiver, during both the initial treatment and the maintenance phase. Three studies limited or excluded caregiver involvement (Chen et al. [23, 24]; Foissac et al. [29] and Jensen et al. [31]).

### 3.4 Quality Review

Risk of bias varied across the randomised trials, and study quality varied across the uncontrolled designs. Among the nine articles reporting the six randomised controlled trials, two [25, 26] were rated at overall ‘low risk’ of bias. Four [23, 24, 27, 29] raised ‘some concerns’ based on predefined judgement algorithms [18], and three [21, 22, 28] were rated at overall ‘high risk’. The two uncontrolled pre‐post studies were rated as ‘fair quality’ [30, 31]. A comprehensive overview of the quality assessment ratings for all articles can be found in Figures S1 and S2, respectively.

### 3.5 Effects of Teleintervention Maintenance Programs

In our comprehensive review of teleintervention maintenance programmes, various health outcomes were investigated, which are discussed below. The main results are described in the following tables.

1. Behaviour Change

Outcomes related to behaviour change included diet and physical activity. Eating behaviour was investigated in four of the eight included studies (Table 2); significant improvements were reported in three (Chen et al. [23, 24]; Nguyen et al. [25, 26]; Straker et al. [30]), whereas Foissac et al. [29] found no significant improvement in the intervention group. Physical activity was assessed in five studies, although outcomes were reported in only four (Table 3): significant between‐group or pre–post improvements were found in three (Chen et al. [23, 24], Straker et al. [30] and Nguyen et al. [25, 26]) and no significant change in one (Foissac et al. [29]). Schiel et al. [28] measured physical activity objectively by sensor‐based mobile‐phone accelerometry and used these data to set individual activity goals during aftercare, but did not report changes in physical activity over the follow‐up period.

- Diet (Vegetables/Fruit Servings, Soda Drinks)

b Physical Activity and Screen Time

2 Psychosocial Outcomes

Psychosocial outcomes were assessed in three of the eight included studies, covering self‐efficacy, quality of life, mental health, self‐perception and body‐shape satisfaction (Table 4). Only one study demonstrated a significant advantage of the teleintervention over its comparator: dietary self‐efficacy (*z* = 5.05, *p* = 0.001; large effect) and physical‐activity self‐efficacy (*z* = 2.75, *p* = 0.006) increased in the intervention group relative to control at 6 months (Chen et al. [21, 22]). The remaining two reported improvements that were not attributable to the digital component. In one, body‐shape satisfaction (+0.43, 95% CI 0.20–0.65), subjective social status (+1.26, 0.86–1.66) and global self‐worth (+0.20, 0.09–0.32) improved pre–post across both arms, with most Harter self‐perception domains improving, while the additional therapeutic contact conferred no benefit and was associated with lower perceived athletic competence (Nguyen et al. [25, 26]). In the other, self‐reported quality of life improved significantly in both the remote and traditional arms (71.7 → 77.5 and 74 → 80.3, respectively, both *p* < 0.001) with no significant between‐group difference, and parent‐reported quality of life did not change (Foissac et al. [29]). Two further studies collected psychosocial data that could not be synthesised: one assessed depression, anxiety and stress (DASS‐21) with no significant between‐ or within‐group change (Abraham et al. [27]) and one reported its psychosocial and quality‐of‐life outcomes in a separate publication (Straker et al. [30]). Psychosocial improvement during the maintenance phase was therefore common but rarely attributable to tele‐delivery specifically.

3 Healthy Lifestyle: Self‐Monitoring and Engagement With the Teleintervention

Self‐management behaviours, operationalised as self‐monitoring and engagement with the intervention tools, were addressed by all eight included studies, but quantified in only four (Table 5). Where engagement was quantified, initial uptake was high: 75% of adolescents used the tracking app at least several times weekly (Chen et al. [23, 24]), 87.5% read the internet curriculum, and 71% completed all lessons (Abraham et al. [27]), and diet was self‐monitored on 48.3% of days during the in‐person phase (Jensen et al. [31]). Sustained engagement was consistently lower: booster‐session attendance fell from 69% to 31% with a median message reply rate of 12% (Nguyen et al. [25, 26]), and only four parents engaged with the curriculum despite high adolescent uptake (Abraham et al. [27]). One study tested this decline statistically and found that engagement fell significantly once the in‐person component was withdrawn: diet self‐monitoring decreased from 48.3% to 16.6% of days and physical‐activity self‐monitoring from 14.6% to 4.6% during the electronic‐only maintenance phase (Jensen et al. [31]: *t* (15) = 5.68, *p* < 0.001, *d* = 0.46; *t* (15) = 3.67, *p* = 0.002, *d* = 0.38), with no significant correlation between self‐monitoring and change in BMI z‐score. Four studies did not quantify adherence to the self‐monitoring component, either reporting it descriptively (Schiel et al. [28]) or not analysing it at all (Straker et al. [30]; Foissac et al. [29]; Garza et al. [21]/Rhyu and Park [22]), limiting conclusions about the relationship between engagement and outcomes.

4 Technology Use, Feasibility, Adherence, Acceptance

Feasibility, adherence and acceptability were addressed in seven of the eight included studies (Table 6); the remaining study reported no formal feasibility data, noting that a separate process evaluation was underway (Straker et al. [30]). Delivery was generally feasible across modalities: telephone, text message, internet curriculum and mobile application. The global acceptability was consistently high: all adolescents in one study would recommend the programme (100%; Chen et al. [23, 24]), as would 87% of adolescents and 97% of parents in another (Nguyen et al. [25, 26]); 81%–100% of participants rated the internet curriculum, weekly text messages and goal‐setting as useful or very useful (Abraham et al. [27]); overall satisfaction reached 20.33 of a maximum 22 on the Client Satisfaction Questionnaire (Jensen et al. [31]); and high adherence with a positive subjective experience of telemedical support was reported as a strength of the intervention (Schiel et al. [28]).

These global ratings, however, concealed a consistent preference for human contact. In the only study to interview participants about the electronic‐only maintenance phase specifically, 73.3% stated that they preferred meeting in a group over the electronic‐only intervention and 53.3% found the self‐monitoring application ‘tedious’ and ‘difficult to use’, while text messages from study staff, the element retaining human involvement, were viewed positively by 73.3% (Jensen et al. [31]). Feasibility was also resource‐dependent: three studies reported substantial staff input, including approximately 2 h per week of research‐assistant time for personalised reminders (Chen et al. [23, 24]; Abraham et al. [27]) and weekly 10–20 min calls delivered by a single trained investigator over 10 months (Garza et al. [21]/Rhyu and Park [22]). The most clearly quantified benefit was a reduction in healthcare burden and cost rather than improved clinical outcomes: remote monitoring halved follow‐up visits (median 5–2, *p* < 0.0001) at approximately 2.5 times lower cost per patient per year, with equivalent weight control (Foissac et al. [29]).

5 Health Outcomes

- Metabolic Profiles, Cardiovascular Function, Body Composition

Health outcomes were assessed in six of the eight included studies (Table 7). The clearest aftercare effect was one of maintenance: in the only study with a camp‐only comparator, telephone aftercare preserved improvements in lipid profile, cardiovascular fitness and body fat that reverted without it (Garza et al. [21]; Rhyu and Park [22]). Otherwise findings were limited: one study reported a reduction in diastolic blood pressure (Chen et al. [23, 24]), one reported mixed changes including an unfavourable decrease in HDL cholesterol (Nguyen et al. [25, 26]), and two reported predominantly null metabolic and cardiovascular findings (Abraham et al. [27]; Foissac et al. [29]). A sixth study assessed a comprehensive metabolic and cardiovascular panel, including carotid intima‐media thickness, 24‐h ambulatory blood pressure, bioelectrical impedance analysis and fasting laboratory parameters, but reported only baseline and discharge comparisons between groups, with no significant differences (e.g., intima‐media thickness 0.56 ± 0.09 vs. 0.56 ± 0.08 mm, *p* = 0.93; systolic blood pressure 136.9 ± 12.7 versus 133.2 ± 10.7 mmHg, *p* = 0.28); changes over the 12‐month aftercare period were not reported (Schiel et al. [28]). Across the evidence base, metabolic and cardiovascular parameters therefore remained largely unchanged, and only one study demonstrated an improvement attributable to the teleintervention itself.

b Weight‐Related Outcomes (BMI z‐Scores, BMI SDS and BMI)

Weight‐related outcomes were reported by all eight included studies, using BMI z‐score or BMI‐SDS in six studies and raw BMI in two that reported no age‐ and sex‐adjusted metric (Garza et al. [21]/Rhyu and Park [22]; Abraham et al. [27]) (Table 8). Findings differed according to the phase to which the change was attributable. Only one study demonstrated a weight benefit attributable to the teleintervention relative to a comparator (Chen et al. [23, 24]: BMI z‐score time × group *p* = 0.001). In two studies, weight reductions achieved during the preceding intensive treatment were retained over the aftercare period, whereas comparison groups without such aftercare returned towards baseline (Garza et al. [21]/Rhyu and Park [22]; Schiel et al. [28]). One study reported a pre–post reduction across both arms to which the telephone/electronic adjunct contributed no additional benefit (Nguyen et al. [25, 26]). In the remaining studies, reductions were either not sustained (Straker et al. [30]: significant at 3 and 6 months but not at 12 months; Jensen et al. [31]: gains reverted to baseline during the electronic‐only phase) or not significant (Abraham et al. [27]; Foissac et al. [29]: mean BMI z‐score change −0.21 remote vs. −0.19 traditional, *p* = 0.87). Taken together, teleintervention aftercare was more consistently associated with stabilisation of previously achieved weight reductions than with further weight loss.

Our findings underscore the role of teleintervention aftercare programs in sustaining the achieved progress in effectively influencing individuals' lifestyles, particularly in daily decision‐making such as dietary habits, and behaviour adjustments such as managing screen time. In the context of lifestyle modifications, four studies resulted in significant improvements in dietary patterns [23, 24, 25, 26, 29, 30], and two studies in significant improvements in physical activities and leisure time [23, 24, 25, 26]. In addition, teleintervention aftercare programs were associated with improvements in enhanced psychosocial outcomes, and these benefits could be confirmed in significant results in at least three studies [23, 24, 25, 26, 27, 29]. Moreover, four studies provided valuable insights into the feasibility [23, 27], adherence [28] and satisfaction [31] of the teleintervention aftercare programs. Furthermore, two studies demonstrated significant improvements in metabolic profiles [21, 25, 26], while two others showed enhancements in cardiovascular health [23, 25, 26]. Additionally, one study indicated improvements in cardiovascular function [21], one reported significant reductions in body fat percentage [21, 22], and one a significant reduction in fat distribution [25, 26].

The BMI is still a key health indicator in determining health outcomes and how interventions aimed at influencing it. Our analysis of 6 RCTs and 2 pre‐post studies (without a control group) shows that four studies reported significant BMI reductions [23, 24, 25, 26, 28], four studies observed significant decreases in BMI z‐scores [23, 25, 26, 29, 30] and one study noted a significant reduction in BMI‐SDS [28].

## 4 Discussion

In this systematic review, we synthesised evidence on long‐term teleintervention aftercare following a first structured paediatric obesity treatment, with a deliberate focus on outcomes beyond BMI. In contrast to the earlier meta‐analysis of maintenance interventions [8], which was not specific to digital delivery and centred on anthropometric endpoints, this review maps the post‐evidence on tele‐delivered aftercare and its effects on psychosocial and behavioural outcomes.

Across the included studies, teleintervention aftercare showed promising effects on several non‐weight outcomes, including dietary behaviours and screen‐time‐related outcomes, and improvements in psychosocial parameters in a subset of trials. In addition, multiple studies reported feasibility, adherence, and satisfaction signals that are highly relevant for long‐term implementation. At the same time, most interventions continued to frame ‘success’ primarily in BMI‐related terms, meaning that the strongest and most frequently reported effects reflect the weight‐centred design of the underlying trials and highlight a persistent gap between guideline ideals (holistic outcomes) and what is most commonly measured in rehabilitation practice.

Across studies, one clear cross‐study signal was that effective digital aftercare is rarely ‘digital only.’ Programs that demonstrated meaningful and sustained change typically included a human component (e.g., clinician contact, case managers, or structured supportive relationships), suggesting that accountability and therapeutic alliance may be central mechanisms for maintaining behaviour change in youth.

This maintenance pattern should be interpreted cautiously, as the studies most clearly demonstrating it (Garza et al. [21] and Park et al. [22]; Schiel et al. [28]) were also those at highest risk of bias.

A consistent pattern was the role of the family system. Studies that limited caregiver involvement (e.g., Chen et al. [23, 24]; Foissac et al. [29]; Jensen et al. [31]) tended to show weaker or less sustained effects, consistent with the role of caregivers in maintaining behaviour change in this age group.

Across studies, feasibility and acceptability were consistently high where formally assessed: 87%–100% of adolescents would recommend the programmes, and satisfaction ratings were favourable (Client Satisfaction Questionnaire 20.33 of 22 in one trial). Yet three patterns temper this apparent success. First, adherence to the digital component declined markedly over time: in the only study to test this statistically, self‐monitoring fell from 48.3% of days during the supported phase to 16.6% once contact was withdrawn [31], and booster‐session attendance in another trial dropped from 69% to 31% [27, 28]. Second, high global satisfaction coexisted with a clear preference for human contact: 73.3% of participants in one study preferred group meetings over the electronic‐only phase, and the automated self‐monitoring application was the least‐liked component [31]. Third, the most robustly quantified benefit was not clinical but organisational: remote monitoring halved follow‐up visits (median 5–2, *p* < 0.0001) at roughly 2.5 times lower cost per patient, with equivalent weight control [27]. Feasibility, in other words, was resource‐dependent on the provider side (staff time for reminders and calls) even as it reduced burden on the patient side.

When reviewing the literature, it was often difficult to distinguish a real maintenance phase, as defined in the Introduction, from ongoing primary care augmented by digital tools. Four studies [32, 33, 34, 35] were excluded on this basis, because it remained unclear whether the teleintervention constituted a distinct follow‐up intervention building on a completed initial treatment, or merely an adjunct to continuous care. This same requirement, that digital delivery is an intended feature of a discrete maintenance phase rather than a circumstantial or continuous element, also excluded programmes in which remote delivery was a temporary adaptation to pandemic‐related restrictions. The difficulty of applying this distinction consistently across the literature is itself a finding: the boundary between ‘maintenance’ and ‘ongoing treatment’ is poorly standardised in the healthcare setting, which complicates both synthesis and clinical translation.

These findings carry several implications for the design of future maintenance trials. First, delivery mode matters more than digital sophistication: aftercare that retained human contact (telephone coaching and case‐manager support) preserved treatment gains, whereas an electronic‐only maintenance phase did not, with gains reverting to baseline as engagement collapsed [31]. Automated feedback alone appears insufficient. Second, dose and duration should be pre‐specified and adequate: the studies with the weakest effects had the shortest maintenance phases (~12 weeks) and the least interactive designs [27, 31], suggesting that maintenance support must extend well beyond the immediate post‐treatment period. Third, family involvement warrants deliberate design, particularly for younger children, since programmes that excluded caregivers showed weaker or null effects [29]. Fourth, and most consequentially for the evidence base, future trials should pre‐specify and report behavioural, engagement and maintenance‐specific outcomes rather than defaulting to weight metrics: several included studies collected behavioural, adherence or physiological data that were never analysed or were reported only in separate publications [28, 29, 30] and few isolated the effect of aftercare from that of the preceding intensive treatment. A comparator that received the initial treatment without aftercare, as in the single study with that design [21, 22], is essential to attribute maintenance effects to the aftercare itself.

### 4.1 Strengths and Limitations

This review has several strengths. First, we included the use of a comprehensive search strategy which was developed with the help of a scientific consultant from the field of rehabilitation and aftercare, following the PRISMA guidelines and a research protocol. Second, we adapted the search to the specific requirements of the included four databases and used connected papers (https://www.connectedpapers.com). These efforts minimise the risk that some relevant studies may have been overlooked. Third, screening of abstracts and full‐text articles was performed by three and four reviewers, respectively, and data were extracted independently by four researchers, strengthening the reliability of the extracted data. In this context, multiple reports based on identical study cohorts were treated as single studies during data synthesis to avoid double counting, prevent overestimation of effects and minimise potential bias. Fourth, the selection of search terms and keywords focusing on interventions and programmes delivered as aftercare scalable within community‐based, healthcare, or tertiary care contexts is also a key strength in our approach. We covered both the specialised and everyday aspects of healthcare delivery in the spectrum of aftercare interventions. Moreover, we studied participants in the children and adolescent group in a wide range of ages (6–18 years), which allowed us to retrieve a wide range of studies in the field of paediatric obesity. Furthermore, this is the first review focusing on outcomes other than BMI in teleintervention aftercare programmes. By applying transparent and replicable methods, 11 articles were identified, providing a comprehensive overview of the current state of scientific evidence in aftercare teleintervention programmes and their outcomes in the context of paediatric obesity.

Despite these strengths, the review has several limitations. First, screening decisions were not blinded between reviewers, although each stage was conducted independently before consensus discussion. Second, only eight of the 11 included papers involved unique study populations. Third, the number of six RCTs examining outcomes beyond weight‐related outcomes was limited. Fourth, studies examining the effect of teleintervention aftercare programs for paediatric obesity are (1) limited in number, (2) content, (3) frequency and (4) interaction, and (5) the devices used vary in the studies discussed and are difficult to compare. Fifth, when evaluating aftercare interventions, it's crucial to differentiate between RCTs with control groups receiving usual care without additional aftercare and those with standard care that includes specific elements like routine clinic visits or community health settings. Sixth, the duration of the initial treatment varied between 6 weeks and 3 months, which may have influenced the level of initial success and participant motivation. This distinction affects comparability overall and limits the assessment of effectiveness. Finally, the heterogeneity described above precluded quantitative pooling, and the strength of conclusions is therefore limited to the direction and consistency of effects across studies. A formal GRADE assessment of certainty was not conducted given the small number of heterogeneous studies per outcome; instead, risk of bias was appraised using ROB2 for randomised trials and the NIH quality‐assessment tool for uncontrolled pre‐post studies.

The present systematic review highlights teleintervention aftercare as a practical support for children and adolescents living with overweight or obesity in sustaining progress beyond the initial intensive phase. Aftercare is essential in paediatric obesity because maintenance is often where families struggle most: routines fade, follow‐up becomes inconsistent and weight regain can occur rapidly. Teleintervention offers a scalable way in the healthcare system to maintain behavioural goals while supporting overall well‐being and long‐term health in this chronic condition for a growing vulnerable group.

In the latest trial, evidence suggests that telemonitoring can preserve outcomes even when face‐to‐face resources are limited. Foissac et al. [29] compared in their randomised trial remote app‐based follow‐up with traditional multidisciplinary visits, and remote monitoring produced similar weight outcomes while significantly reducing follow‐up visits, and the traditional pathway was described as ~2.5 times more expensive per patient per year despite comparable results. These findings strengthen the case for teleintervention as a continuity strategy for health systems under strain and for families who cannot reliably attend frequent appointments.

Mobile health (mHealth) is a promising way to deliver weight management in paediatric obesity. However, there is no evidence concerning the costs and effectiveness of current mHealth interventions for weight loss.

Therefore, future research should move beyond the question of whether digital aftercare and approaches ‘are effective’ and instead identify which components work best, for which target group, and in which settings. This includes clarifying the optimal intensity and mode of support (e.g., therapist‐led, peer‐based, or parent‐supported) and determining whether continuous or tapered contact more effectively promotes autonomy and sustained engagement over time. Evaluation frameworks should also adopt a broader definition of success than metrics like weight change alone. Alongside BMI‐related outcomes, studies need to prioritise participant satisfaction, adherence, quality of life, behavioural stability and other determinants of long‐term health and well‐being in chronic disease management^1^.

## Funding

This work was supported by Bundesministerium für Arbeit und Soziales.

## Conflicts of Interest

The authors declare no conflicts of interest.

## Supporting information


**Figure S1:** Quality assessment for RCTs.

**Figure S2:** Quality assessment for Pre‐post studies.

**Table S1:** PRISMA checklist.
**Table S2:** Search strategy.
**Table S3:** Inclusion and exclusion criteria.
**Table S4:** Reasons for excluding the articles not retrieved (*n* = 71).

## Data Availability

The data that supports the findings of this study are available in the Supporting Information of this article.
