# Supplementary material for: Effects of Teleintervention Maintenance Programs for Children (Age 6–12) and Adolescents (Age 13–18) With Overweight or Obesity—Promoting a Healthy Lifestyle: A Systematic Review
Source: Pediatr Obes. 2026 Sep 27;21(10):e70158. doi: 10.1111/ijpo.70158 (PMC13616525; doi:10.1111/ijpo.70158)
Supplement: Supplementary file 1 [file IJPO-21-e70158-s002.pdf]

Effects of teleintervention maintenance programs for children (age 6-12) and adolescents (age 13-18) with overweight or obesity - promoting a healthy lifestyle: a systematic review

Supporting Information

Anna Wittmann[1], Pia Späth[1], Feline Zocher[2], Eckard Nagel[1], Michael Lauerer[1], Magdalena Schellenberg[1]

[1] Institute for Medical Management and Health Sciences, University of Bayreuth, Germany - [2] Deutsche Rentenversicherung Nordbayern, Bayreuth, Germany

Corresponding author: A. Wittmann, M.Sc., Institute for Medical Management and Health Sciences, University of Bayreuth, Prieserstrasse 2, 95444 Bayreuth, Germany.
E-Mail: anna.wittmann@uni-bayreuth.de

Appendix

Supplementary Figure S1: Quality assessment for RCTs

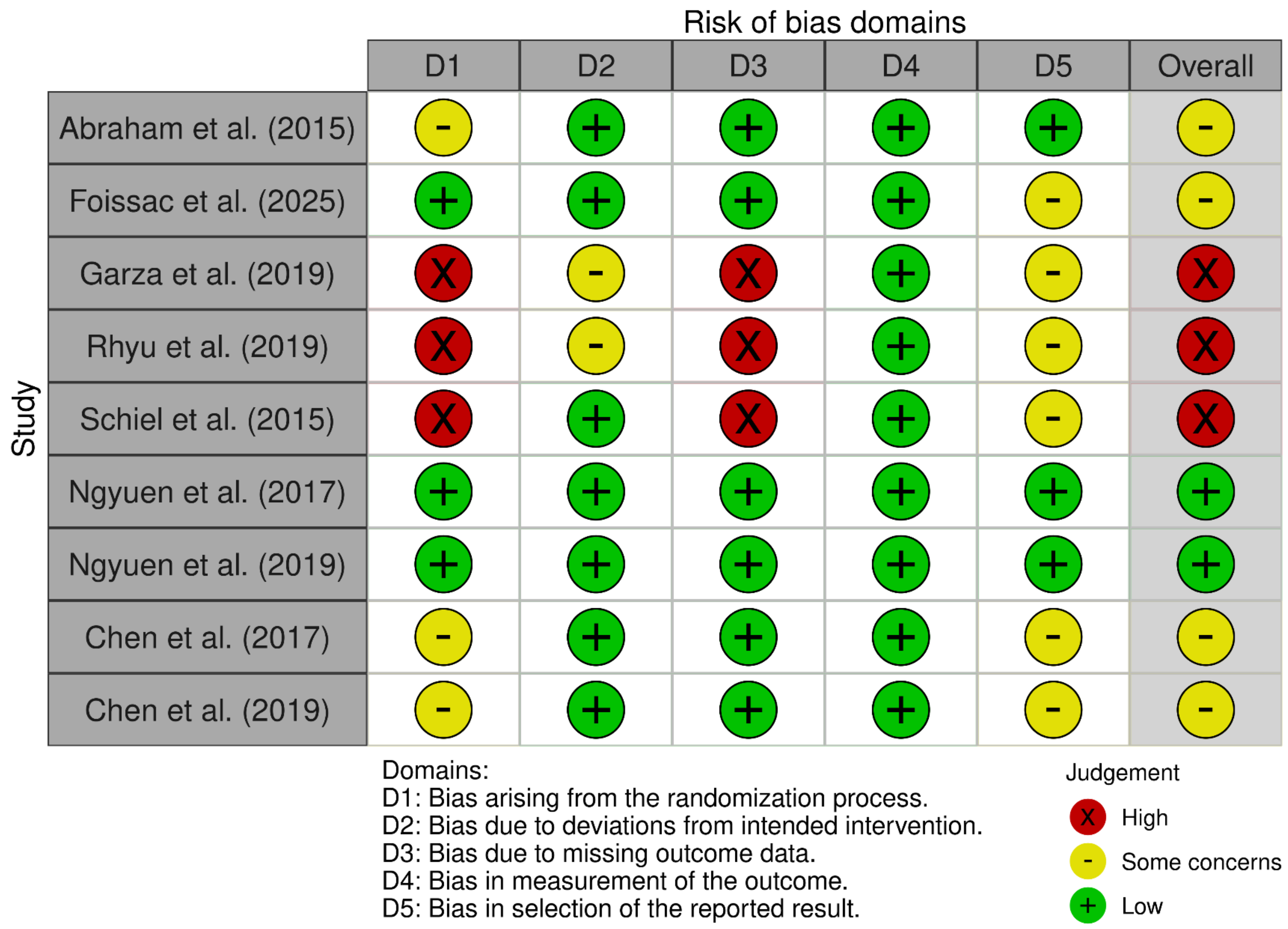
Risk of bias domains
D1 D2 D3 D4 D5 Overall
Study
Abraham et al. (2015) - + + + + -
Foissac et al. (2025) + + + + - -
Garza et al. (2019) X - X + - X
Rhyu et al. (2019) X - X + - X
Schiel et al. (2015) X + X + - X
Ngyuen et al. (2017) + + + + + +
Ngyuen et al. (2019) + + + + + +
Chen et al. (2017) - + + + - -
Chen et al. (2019) - + + + - -
Domains:
D1: Bias arising from the randomization process.
D2: Bias due to deviations from intended intervention.
D3: Bias due to missing outcome data.
D4: Bias in measurement of the outcome.
D5: Bias in selection of the reported result.
Judgement
X High
- Some concerns
+ Low

Supplementary Figure S2: Quality assessment for Pre-post studies

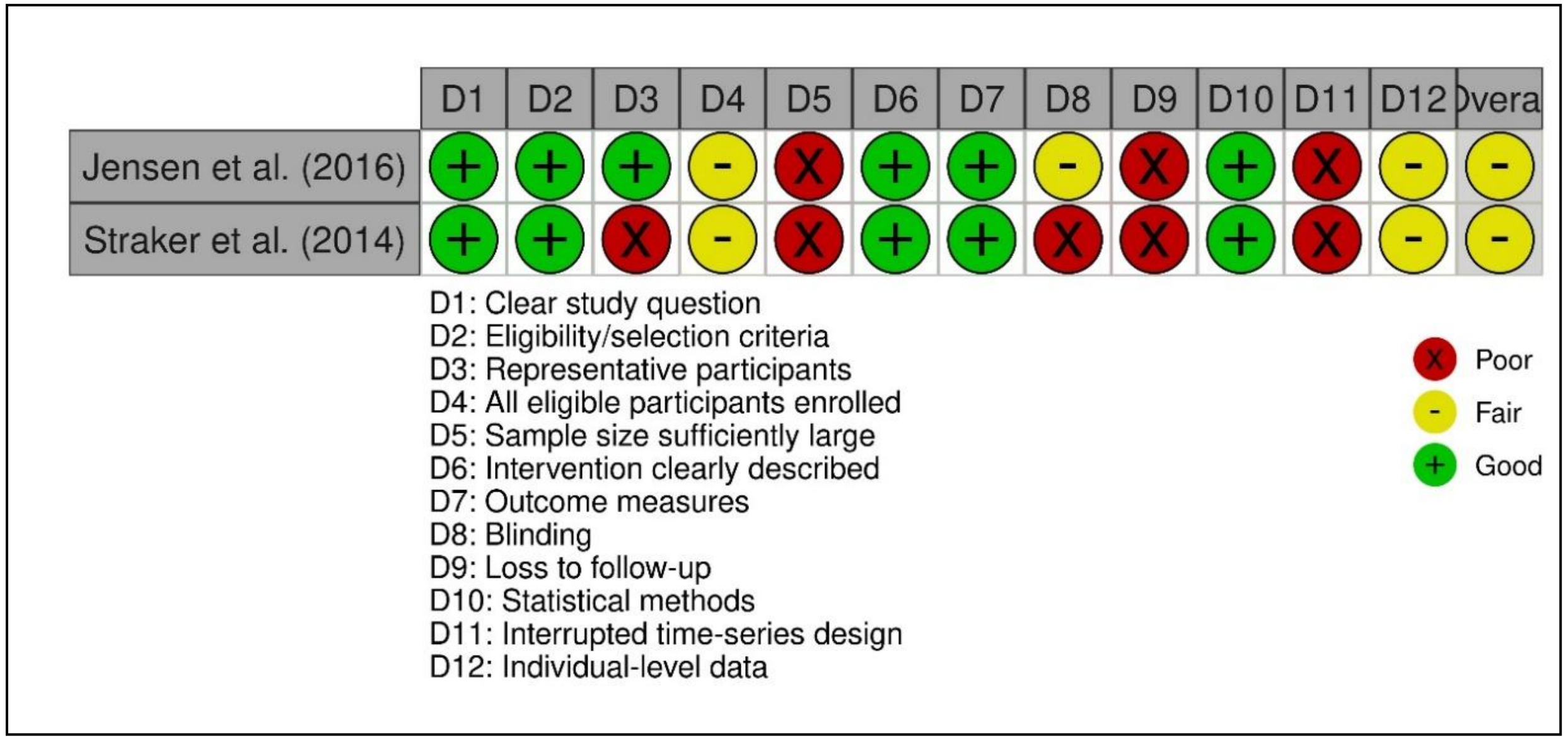

Supplementary Table S1: PRISMA checklist

Section and Topic	Item #	Checklist item	Location where item is reported
**TITLE**			
Title	1	Identify the report as a systematic review.	1
**ABSTRACT**			
Abstract	2	See the PRISMA 2020 for Abstracts checklist.	2
**INTRODUCTION**			
Rationale	3	Describe the rationale for the review in the context of existing knowledge.	3-4
Objectives	4	Provide an explicit statement of the objective(s) or question(s) the review addresses.	5-6
**METHODS**			
Eligibility criteria	5	Specify the inclusion and exclusion criteria for the review and how studies were grouped for the syntheses.	
Information	6	Specify all databases, registers, websites, organisations, reference lists and other sources searched or consulted to identify studies. Specify the	6

Section and Topic	Item #	Checklist item	Location where item is reported
sources		date when each source was last searched or consulted.	
Search strategy	7	Present the full search strategies for all databases, registers and websites, including any filters and limits used.	35
Selection process	8	Specify the methods used to decide whether a study met the inclusion criteria of the review, including how many reviewers screened each record and each report retrieved, whether they worked independently, and if applicable, details of automation tools used in the process.	8
Data collection process	9	Specify the methods used to collect data from reports, including how many reviewers collected data from each report, whether they worked independently, any processes for obtaining or confirming data from study investigators, and if applicable, details of automation tools used in the process.	8
Data items	10a	List and define all outcomes for which data were sought. Specify whether all results that were compatible with each outcome domain in each study were sought (e.g. for all measures, time points, analyses), and if not, the methods used to decide which results to collect.	9
	10b	List and define all other variables for which data were sought (e.g. participant and intervention characteristics, funding sources). Describe any assumptions made about any missing or unclear information.	(n.a.)
Study risk of bias assessment	11	Specify the methods used to assess risk of bias in the included studies, including details of the tool(s) used, how many reviewers assessed each study and whether they worked independently, and if applicable, details of automation tools used in the process.	8-9
Effect measures	12	Specify for each outcome the effect measure(s) (e.g. risk ratio, mean difference) used in the synthesis or presentation of results.	(n.a.)
Synthesis methods	13a	Describe the processes used to decide which studies were eligible for each synthesis (e.g. tabulating the study intervention characteristics and comparing against the planned groups for each synthesis (item #5)).	(n.a.)
	13b	Describe any methods required to prepare the data for presentation or synthesis, such as handling of missing summary statistics, or data conversions.	(n.a.)
	13c	Describe any methods used to tabulate or visually display results of individual studies and syntheses.	(n.a.)
	13d	Describe any methods used to synthesize results and provide a rationale for the choice(s). If meta-analysis was performed, describe the model(s), method(s) to identify the presence and extent of statistical heterogeneity, and software package(s) used.	(n.a.)
	13e	Describe any methods used to explore possible causes of heterogeneity among study results (e.g. subgroup analysis, meta-regression).	(n.a.)
	13f	Describe any sensitivity analyses conducted to assess robustness of the synthesized results.	(n.a.)
Reporting bias assessment	14	Describe any methods used to assess risk of bias due to missing results in a synthesis (arising from reporting biases).	(n.a.)
Certainty assessment	15	Describe any methods used to assess certainty (or confidence) in the body of evidence for an outcome.	(n.a.)
**RESULTS**			
Study selection	16a	Describe the results of the search and selection process, from the number of records identified in the search to the number of studies included in the review, ideally using a flow diagram.	9-10
	16b	Cite studies that might appear to meet the inclusion criteria, but which were excluded, and explain why they were excluded.	26

Section and Topic	Item #	Checklist item	Location where item is reported
Study characteristics	17	Cite each included study and present its characteristics.	40-44
Risk of bias in studies	18	Present assessments of risk of bias for each included study.	30-31
Results of individual studies	19	For all outcomes, present, for each study: (a) summary statistics for each group (where appropriate) and (b) an effect estimate and its precision (e.g. confidence/credible interval), ideally using structured tables or plots.	46-54
Results of syntheses	20a	For each synthesis, briefly summarise the characteristics and risk of bias among contributing studies.	(n.a.)
	20b	Present results of all statistical syntheses conducted. If meta-analysis was done, present for each the summary estimate and its precision (e.g. confidence/credible interval) and measures of statistical heterogeneity. If comparing groups, describe the direction of the effect.	(n.a.)
	20c	Present results of all investigations of possible causes of heterogeneity among study results.	(n.a.)
	20d	Present results of all sensitivity analyses conducted to assess the robustness of the synthesized results.	(n.a.)
Reporting biases	21	Present assessments of risk of bias due to missing results (arising from reporting biases) for each synthesis assessed.	(n.a.)
Certainty of evidence	22	Present assessments of certainty (or confidence) in the body of evidence for each outcome assessed.	(n.a.)
**DISCUSSION**			
Discussion	23a	Provide a general interpretation of the results in the context of other evidence.	23-25
	23b	Discuss any limitations of the evidence included in the review.	26-27
	23c	Discuss any limitations of the review processes used.	(n.a.)
	23d	Discuss implications of the results for practice, policy, and future research.	27-28
**OTHER INFORMATION**			
Registration and protocol	24a	Provide registration information for the review, including register name and registration number, or state that the review was not registered.	6
	24b	Indicate where the review protocol can be accessed, or state that a protocol was not prepared.	6
	24c	Describe and explain any amendments to information provided at registration or in the protocol.	(n.a.)
Support	25	Describe sources of financial or non-financial support for the review, and the role of the funders or sponsors in the review.	(n.a.)
Competing interests	26	Declare any competing interests of review authors.	(n.a.)
Availability of data, code and other materials	27	Report which of the following are publicly available and where they can be found: template data collection forms; data extracted from included studies; data used for all analyses; analytic code; any other materials used in the review.	46-59

Supplementary Table S2: Search strategy

	AND	AND	AND	AND
OR	Adolescent[MeSH Terms]<br>Adolescen*<br>Youth*<br>Young*<br>Juvenile*<br>Boy*<br>Girl*<br>Child[MeSH Terms]<br>Child<br>Kinder<br>Jugend*<br>Jung*<br>Mädchen	obesity[MeSH Terms]<br>obesity<br>obese<br>adiposity[MeSH Terms]<br>adiposity<br>Overweigh*<br>Adipös*<br>Übergewicht*<br>Fettleib*<br>Adipos*	Telemedicine[MeSH Terms]<br>Tele*<br>Digital Technology[MeSH Terms]<br>Digital*<br>Mobile Applications[MeSH Terms]<br>Mobil*<br>Internet*<br>Web*<br>Online*<br>eHealth<br>mHealth<br>App<br>Application<br>Neue Medien<br>Smartphone*<br>Tablet*	aftercare[MeSH Terms]<br>Aftercare*<br>Follow-up care<br>Aftertreat*<br>reha*<br>Nachsorg*
FILTER		AND (2012/01/01:2025/11/04[Date - Publication]) AND (english[Filter] OR german[Filter])		

Supplementary Table S3: Inclusion and exclusion criteria

**Included studies** had to meet the following further inclusion criteria	**Studies had been excluded** when:
I. examining children and adolescents aged 6-18 years, based on the PubMed MeSH definition of children (6-12 years) and adolescents (13-18 years);<br>II. with a BMI>=25, or BMI>p85 for age, or BMI z-score>+1SD;<br>III. should have followed an structured treatment program for overweight or obesity, and<br>IV. duration of the aftercare teleintervention program should be at least a minimum 12 weeks long (regardless of the type of digital intervention);<br>V. report one of the following outcomes: healthy lifestyle parameter, psychosocial outcomes or behaviour change next to weight or BMI;<br>VI. language in either English or German;<br>VII. when children and adolescents are part of the study population as a family in a community-based setting receiving the aftercare intervention, the study will be included if data can be extracted separately for the children and adolescents.	I. not available (no full text or containing only the abstract);<br>II. the study is a systematic review or meta-analysis, qualitative study, cross-sectional study or study protocol;<br>III. primary prevention studies on obesity with non-overweight or children with or adolescents with obesity as the study population;<br>IV. children or adolescents with overweight or obesity due to a secondary or syndromic cause;<br>V. parents as the target group of the aftercare intervention, without addressing children and adolescents separately;<br>VI. teleintervention is part of a multi-intervention programme;<br>VII. target population consists exclusively of infants or young adults (i.e., <6 or >18 years old)

Supplementary Table S4: Reasons for excluding the articles not retrieved (n=71)

**Author**	**Year**	**Title**	**Exclusion reason**	
Gardia, C., Park, K.-S	2012	Effects Of Telephone Intervention On Maintaining Fat Loss And Fitness Level Following Summer Intervention Program	2	Publication type
Lucht, M., et al.	2012	The use of exer-learning games for rehabilitation in spa clinics at home	3	Not available
Tambe, K., et al.	2012	Multidisciplinary management of periocular necrotising fasciitis: a series of 11 patients	1	Off topic
Tayebeh Malek, M., et al.	2012	The association of body mass index with dental caries in an Iranian sample of children	1	Off topic
Uzark, K., et al.	2012	Health-Related Quality of Life in Children and Adolescents With Duchenne Muscular Dystrophy	1	Off topic
Vaidya, R., et al.	2012	Complications of anterior subcutaneous internal fixation for unstable pelvis fractures: a multicenter study	1	Off topic
Patrick, K., et al.	2013	Outcomes of a 12-month technology-based intervention to promote weight loss in adolescents at risk for type 2 diabetes	5	Intervention type
DuBose, K. D., et al.	2014	The Effect of a Telephone-Based Physical Activity Intervention in Obese Adolescents	3	Not available
Riiser, K., et al.	2014	The outcomes of a 12-week Internet intervention aimed at improving fitness and health-related quality of life in overweight adolescents: the Young & Active controlled trial	5	Intervention type
Nourse, S. E., et al.	2015	Live Video Diet and Exercise Intervention in Overweight and Obese Youth: Adherence and Cardiovascular Health	5	Intervention type
Pbert, L., et al.	2016	Use of a FITLINE to Support Families of Overweight and Obese Children in Pediatric Practices	5	Intervention type
Slusser, W., et al.	2016	Multidisciplinary Pediatric Obesity Clinic via Telemedicine Within the Los Angeles Metropolitan Area: Lessons Learned	5	Intervention type
Stumm, G., et al.	2016	Long-Term Follow-Up of the Telemonitoring Weight-Reduction Program "Active Body Control"	6	Wrong age
Bohlin, A., et al.	2017	Childhood obesity treatment: telephone coaching is as good as usual care in maintaining weight loss - a randomized controlled trial	6	Wrong age
Dorow, M., et al.	2017	[Willingness of Patients with Obesity to Use New Media in Rehabilitation Aftercare]	6	Wrong age
Podina, I. R., et al.	2017	An evidence-based gamified mHealth intervention for overweight young adults with maladaptive eating habits: study protocol for a randomized controlled trial	2	Publication type
Cattivelli, R., et al.	2018	ACTonHEALTH study protocol: promoting psychological flexibility with activity tracker and mHealth tools to foster healthful lifestyle for obesity and other chronic health conditions	2	Publication type
Mameli, C., et al.	2018	Combined use of a wristband and a smartphone to reduce body weight in obese children: randomized controlled trial	5	Intervention type
Rivera, J., et al.	2018	User-Centered Design of a Mobile App for Weight and Health Management in Adolescents With Complex Health Needs: Qualitative Study	4	Study type
Wulff, H. and Wagner, P.	2018	Media Use and Physical Activity Behaviour of Adolescent Participants in Obesity Therapy: Impact Analysis of Selected Socio-Demographic Factors	5	Intervention type
Wulff, H. and Wagner, P.	2018	The Use of Social Networks and Physical Activity Patterns of Adolescents in Obesity Therapy - A Sociodemographic Stratified Analysis	5	Intervention type
Fang, Y., et al.	2019	Methodology of an exercise intervention program using social incentives and gamification for obese children	5	Intervention type

Karmali, S., et al.	2019	Coaching and/or education intervention for parents with overweight/obesity and their children: study protocol of a single-centre randomized controlled trial	5	Intervention type
Bartosiewicz, A., et al.	2020	Children's Body Mass Index Depending on Dietary Patterns, the Use of Technological Devices, the Internet and Sleep on BMI in Children	5	Intervention type
Johansson, L., et al.	2020	A novel interactive mobile health support system for pediatric obesity treatment: a randomized controlled feasibility trial	4	Study type
Meinert, E., et al.	2020	Acceptability and Usability of the Mobile Digital Health App NoObesity for Families and Health Care Professionals: Protocol for a Feasibility Study	2	Publication type
Naets, T., et al.	2020	Adherence and barriers in e-health self-control training for enhancing childhood multidisciplinary obesity treatment	6	Wrong age
Yoong, S. L., et al.	2020	Child-level evaluation of a web-based intervention to improve dietary guideline implementation in childcare centers: a cluster-randomized controlled trial	5	Intervention type
Chew, C. S. E., et al.	2021	Use of a Mobile Lifestyle Intervention App as an Early Intervention for Adolescents With Obesity: Single-Cohort Study	5	Intervention type
Likhitweerawong, N., et al.	2021	Effectiveness of mobile application on changing weight, healthy eating habits, and quality of life in children and adolescents with obesity: a randomized controlled trial	5	Intervention type
Lim, C., et al.	2021	Design, Implementation, and Examination of a Remote Patient Monitoring System for Pediatric Obesity: Protocol for an Open Trial Pilot Study	2	Publication type
Majaj, R., et al.	2021	Physiological Responses to Active Video Games Compared to Treadmill Walking and TV Watching in Obese Children and Adolescents	5	Intervention type
Pecoraro, P., et al.	2021	A telehealth intervention for ensuring continuity of care of pediatric obesity during the CoVid-19 lockdown in Italy	6	Wrong age
Braddock, A., et al.	2022	A Longitudinal Effectiveness Study of a Child Obesity Electronic Health Record Tool	6	Wrong age
Eisenburger, N., et al.	2022	Short report: Weight management of children and adolescents with obesity during the COVID-19 pandemic in Germany	5	Intervention type
Moravcová, K., et al.	2022	Comparing Digital Therapeutic Intervention with an Intensive Obesity Management Program: Randomized Controlled Trial	5	Intervention type
Poulsen, M. N., et al.	2022	Family-Based Telehealth Initiative to Improve Nutrition and Physical Activity for Children With Obesity and Its Utility During COVID-19: A Mixed Methods Evaluation	5	Intervention type
Putter, K. C., et al.	2022	Perceptions of a family-based lifestyle intervention for children with overweight and obesity: a qualitative study on sustainability, self-regulation, and program optimization	5	Intervention type
Salahshoornezhad, S., et al.	2022	Effect of a multi-disciplinary program on anthropometric and biochemical parameters in obese and overweight elementary school girls: A randomized clinical trial	5	Intervention type
Siegel, R., et al.	2022	Families Chose In-Person Visits over Telehealth for Pediatric Weight Management during the COVID-19 Pandemic	5	Intervention type
Strempfl, J., et al.	2022	Impact of self-determination theory in a physiotherapeutic training : A pilot-study on motivation for movement of obese adolescents	1	Off topic
Apich, G.	2013	Conservative lymphedema therapy - Lymphological rehabilitation treatment. [German]	1	Off topic
Eriks-Hoogland, I., et al.	2013	Acromioclavicular joint arthrosis in persons with spinal cord injury and able-bodied persons	1	Off topic
Espeland, M. A., et al.	2013	Intensive Weight Loss Intervention in Older Individuals: Results from the Action for Health in Diabetes Type 2 Diabetes Mellitus Trial	1	Off topic
Nişancı Kılınç, F. and Çağdaş, D. N.	2013	Diet and physical activity interventions do have effects on body composition and metabolic syndrome parameters in overweight and obese adolescents and their mothers	1	Off topic
Oliver, E., et al.	2013	An electronic system (PDA) to record dietary and physical activity in obese adolescents; data about efficiency and feasibility	5	Intervention type

Sartorio, A., et al.	2013	GH Responses to 2 Consecutive Bouts of Respiratory Muscle Endurance Training in Obese Adolescents and Adults	1	Off topic
Selewski, D. T., et al.	2013	Promising insights into the health related quality of life for children with severe obesity	1	Off topic
Wright, J. A., et al.	2013	Randomized trial of a family-based, automated, conversational obesity treatment program for underserved populations	5	Intervention type
Chang, M. L., et al.	2014	Encouraging obese students with intellectual disabilities to engage in pedaling an exercise bike by using an air mouse combined with preferred environmental stimulation	1	Off topic
Mortazavi, B., et al.	2014	Near-realistic mobile exergames with wireless wearable sensors	1	Off topic
Quintiliani, L. M., et al.	2014	Enhancing physical and social environments to reduce obesity among public housing residents: rationale, trial design, and baseline data for the Healthy Families study	5	Intervention type
Windham, M. E., et al.	2014	Teens Talk Healthy Weight: the impact of a motivational digital video disc on parental knowledge of obesity-related diseases in an adolescent clinic	5	Intervention type
De Miguel-Etayo, P., et al.	2015	BODY COMPOSITION CHANGES DURING A MULTIDISCIPLINARY TREATMENT PROGRAMME IN OVERWEIGHT ADOLESCENTS: EVASYON STUDY	1	Off topic
Lee, H. and Kim, Y.	2015	Effects of an Obesity Intervention Integrating Physical Activity and Psychological Strategy on BMI, Physical Activity, and Psychological Variables in Male Obese Adolescents	1	Off topic
Oreskovic, N. M., et al.	2016	Design and rationale of the STRIVE trial to improve cardiometabolic health among children and families	5	Intervention type
Gesser, u. et al.	2016	Langzeiteffekte der Adipositastherapie _ internetbasierte, ärztliche Nachsorge mit dem Online-Portal _rehasonanz.de	6	Wrong age
Barnes, R. D., et al.	2017	Back to Basics? No Weight Loss from Motivational Interviewing Compared to Nutrition Psychoeducation at One-Year Follow-Up	5	Intervention type
Bennett, E. A., et al.	2017	Treatment of Obesity Among Youth With Intellectual and Developmental Disabilities: An Emerging Role for Telenursing	5	Intervention type
da Silveira Campos, R. M., et al.	2017	Homeostasis Model Assessment-Adiponectin: the role of different types of physical exercise in obese adolescents	1	Off topic
Armstrong, S., et al.	2018	Texting Motivational Interviewing: A Randomized Controlled Trial of Motivational Interviewing Text Messages Designed to Augment Childhood Obesity Treatment	6	Wrong age
Bruñó, A., et al.	2018	Home-exercise Childhood Obesity Intervention: A Randomized Clinical Trial Comparing Print Versus Web-based (Move It) Platforms	5	Intervention type
Eisenburger, N. et al.	2022	Short report: Weight management of children and adolescents with obesity during the COVID-19 pandemic in Germany	5	Intervention type
Calcaterra, V., et al.	2023	Effects of Endurance Exercise Intensities on Autonomic and Metabolic Controls in Children with Obesity: A Feasibility Study Employing Online Exercise Training	5	Intervention type
Chew, H. S. J., et al.	2023	Essential elements of weight loss apps for a multi-ethnic population with high BMI: a qualitative study with practical recommendations	4	Study type
Mannarino, S., et al.	2023	Benefits in Cardiac Function from a Remote Exercise Program in Children with Obesity	5	Intervention type
Afsar, M. et al.	2023	Body-Worn Sensors for Recognizing Physical Sports Activities in Exergaming via Deep Learning Model	1	Off topic
Giuseppina, R. et al.	2024	Effectiveness of Smartphone App for the Treatment of Pediatric Obesity: A Randomized Controlled Trial	5	Intervention type

Ahnert, J. et al	2025		Telemedical Franconian Obesity Aftercare Therapy (Tele-FANT) Results of a Formative Evaluation	6	Wrong age
Calcaterra. V. et al.	2025		Improving cardiometabolic health in children and adolescents with obesity: a comparison between in-person and virtual supervised training	5	Intervention type
Hagman, E. et al.	2025		Long-term results of a digital treatment tool as an add-on to pediatric obesity lifestyle treatment: a 3-year pragmatic clinical trial	6	Wrong age

**Reasons for excluding**	
1 = Off topic	17
2 = Publication type: no article, no monograph, no anthology article, not published (means: study protocol, abstract, poster, etc.)	5
3 = Not available	2
4 = Study type: no primary study, a meta-analysis, a systematic review, qualitative study	3
5 = Intervention type: no aftercare, no curation after rehabilitation, no intervention in terms of follow-up treatment, no intervention in terms of maintenance	34
6 = Wrong age	10
